# Dietary Keratan Sulfate from Shark Cartilage Modulates Gut Microbiota and Increases the Abundance of *Lactobacillus* spp.

**DOI:** 10.3390/md14120224

**Published:** 2016-12-08

**Authors:** Qingsen Shang, Qinying Li, Meifang Zhang, Guanrui Song, Jingjing Shi, Hao Jiang, Chao Cai, Jiejie Hao, Guoyun Li, Guangli Yu

**Affiliations:** 1 Key Laboratory of Marine Drugs, Ministry of Education, School of Medicine and Pharmacy, Ocean University of China, Qingdao 266003, China; shangqingsen@163.com (Q.S.); liqinying2010@163.com (Q.L.); zhangmeifang96@163.com (M.Z.); songguanrui122@163.com (G.S.); jingjshi@126.com (J.S.); haojiang833@163.com (H.J.); caic@ouc.edu.cn (C.C.); 2009haojie@ouc.edu.cn (J.H.); 2 Shandong Provincial Key Laboratory of Glycoscience and Glycotechnology, School of Medicine and Pharmacy, Ocean University of China, Qingdao 266003, China; 3 Laboratory for Marine Drugs and Bioproducts of Qingdao National Laboratory for Marine Science and Technology, Qingdao 266003, China

**Keywords:** keratan sulfate, gut microbiota, modulation, *Lactobacillus* spp., shark cartilage

## Abstract

Keratan sulfate (KS) represents an important family of glycosaminoglycans that are critical in diverse physiological processes. Recently, accumulating evidence has provided a wealth of information on the bioactivity of KS, which established it as an attractive candidate for drug development. However, although KS has been widely explored, less attention has been given to its effect on gut microbiota. Therefore, given that gut microbiota plays a pivotal role in health homeostasis and disease pathogenesis, we investigated here in detail the effect of KS on gut microbiota by high-throughput sequencing. As revealed by heatmap and principal component analysis, the mice gut microbiota was readily altered at different taxonomic levels by intake of low (8 mg/kg) and high dosage (40 mg/kg) of KS. Interestingly, KS exerted a differing effect on male and female microbiota. Specifically, KS induced a much more drastic increase in the abundance of *Lactobacillus* spp. in female (sixteen-fold) versus male mice (two-fold). In addition, combined with alterations in gut microbiota, KS also significantly reduced body weight while maintaining normal gut homeostasis. Altogether, we first demonstrated a sex-dependent effect of KS on gut microbiota and highlighted that it may be used as a novel prebiotic for disease management.

## 1. Introduction

Keratan sulfate (KS) is a glycosaminoglycan (GAG) that is widely distributed in the extracellular matrices of animal tissues such as bone, cartilage and cornea [1,2]. As a liner polymer mainly composed of β-1,3-linked galactose (Gal) and β-1,4-linked *N*-acetylglucosamine (GlcNAc), KS stands out as the only type of GAGs that does not bear an uronic acid residue [2]. However, KS is still recognized as an acidic GAG. This is because KS is highly sulfated, with sulfation usually occurs at C-6 position of both Gal and GlcNAc [1]. Currently, two types of KS have been identified, namely KS I and KS II [2]. KS I is *N*-linked to the asparagine resides in protein and is usually found in corneal and the epidermis of animal skin (for example, fibromodulin) [1,3,4]. In contrast, KS II is *O*-linked to the serine and threonine residues in protein and is much more often detected in cartilage [1,2].

As a functional macromolecule, KS has been comprehensively studied [1]. For example, KS is extremely crucial for maintaining the proper hydration levels of corneal and cartilage [1,3]. Additionally, KS also actively participates in the development of neural system [5,6]. Much more recently, KS was evidenced to be involved in the microbial infection process of corneal stroma and the progress of experimental autoimmune neuritis [1,7]. Taken together, KS plays a biologically important role in various physiological processes, and, as a promising drug candidate, it has been developed as an active ingredient in different pharmaceutical formulations [1]. However, unfortunately, up to now, the commercial utilization of KS, for example, as a medical component in eye drops, is largely confined to its hydration properties [1,2]. Therefore, it is highly desirable that novel bioactivities of KS could be identified to benefit its industrial application as a new therapeutic drug molecule from marine resources.

Gut microbiota is a newly discovered “metaorgan” that has fundamental roles in almost every aspect of host physiology [8,9,10]. Recently, disruption of gut microbiota, often termed as gut dysbiosis, has been illustrated to be firmly linked to various diseases including obesity [11,12], diabetes [12,13,14], colorectal cancer [15,16,17], Crohn’s disease [18,19], and ulcerative colitis [20,21]. Therefore, in this context, restoring the disrupted gut microbiota represents a promising therapeutic strategy for the management of gut microbiota-related diseases [22,23,24]. For this purpose, several medicinal approaches have been successfully developed, and among these, the use of prebiotics has been recognized as one of the most effective and commercially available options for the treatment of gastrointestinal dysbiosis [25,26,27].

Prebiotics are defined as microbiota accessible carbohydrate that could beneficially modulate gut microbiota by increasing the abundance of *Lactobacillus* spp. and *Bifidobacterium* spp. [25,26]. Previously, Turroni et al. demonstrated that host-derived GAGs can be used as nutrient resources for *B. bifidum* [28]. This study shed new light on the use of GAGs as novel source of prebiotics. Recently, heparin and chondroitin sulfate have been identified as potential new prebiotics that could beneficially modulate the intestinal microbiota and stimulate the growth of *Lactobacillus* spp. in the gut of rats [29] and mice [30]. Given that both heparin and chondroitin sulfate are members of the GAG family [31,32], and also in light of the fact that KS cannot be digested by gastric and intestinal enzymes after oral administration [1,2], we extrapolate that KS, as another member of the GAG family, could also modulate the gut microbiota and promote the growth of *Lactobacillus* spp. In the present study, to verify this hypothesis, we analyzed the gut microbiota of Kunming mice after low-dose and high-dose of KS treatment by high-throughput sequencing. As expected, oral intake of KS induced a dramatic change in the composition of the gut microbiota in both male and female mice. However, interestingly, the prebiotic effect of KS was observed to be much more pronounced in female than in male mice. Collectively, our study indicates that KS can be used as a new prebiotic candidate and highlights that potential therapy should be tailored to the individual patient when KS is used as a prebiotic ingredient.

## 2. Results and Discussion

### 2.1. Intake of KS Profoundly Changed the Structure of Gut Bacterial Community in Both Male and Female Mice

The gut microbiota is an extremely dense and diverse microbial community which plays a pivotal role in both health homeostasis and disease pathogenesis [8,9,22]. Given that dietary fiber has been successfully used to manipulate diseases for years by modulating gut microbiota [33,34,35,36], it is, therefore, of interest to elucidate whether KS as a non-digestible polysaccharide could confer beneficial effects by targeting the gut microbes. In the present study, we illustrated in detail the modulations of gut microbiota by low-dosage and high-dosage of KS treatment. Intriguingly, we found that dietary KS significantly increased the abundance of *Lactobacillus* spp. in Kunming mice. Our results rationalize the commercial utilization of KS as a novel prebiotic substrate and will benefit the development of KS as a new generation of marine drug.

During the experiment, no significant clinical symptoms were observed and all mice remained in good health. To fully demonstrate the effect of KS on commensal bacteria, high-throughput sequencing covering the V3-V4 hypervariable regions of 16S rRNA was applied. The Good’s coverage (>99%) indicates a sufficient sequencing depth for all samples (Table 1). After the quality control process, a total of 148,704 high-quality sequencing reads was recovered for downstream analysis (Table 1). Based on these data, similar sequences with 97% cutoff were combined into operational taxonomic units (OTUs) (Table 1). To estimate the gut microbial community richness and diversity, we calculated the Ace, Chao1, Shannon, and Simpson indices. As indicated in Table 1, low-dose and high-dose KS both induced detectable changes in community richness and diversity in male and female mice. However, these changes were more obvious in female groups than in male groups (Table 1). For example, the Shannon indices in female mice shifted from 4.39 to 4.24 and 4.45 in KSLF and KSHF groups, respectively, while in contrast, the Shannon indices were almost unchanged by KS treatment in male mice (Table 1). Taken together, we tentatively put forward that dietary KS may exert a sex-dependent effect on the composition of gut microbiota in Kunming mice.

**Figure 1:**
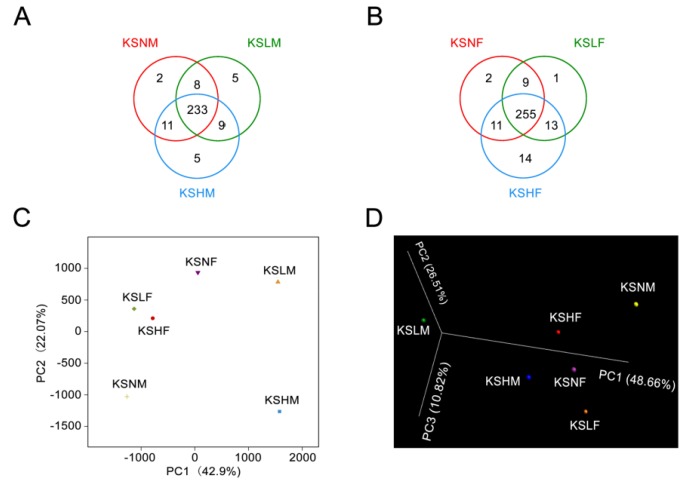
Response of the gut microbiota to KS treatment. Venn diagram representation of shared/unique OTUs in the gut microbiota of male (**A**) and female (**B**) mice; PCA score plot of the gut microbiota in all mice groups (**C**); 3D-PCoA of the gut microbiota-based weighted UniFrac metric (**D**).

To validate this hypothesis, we constructed a Venn diagram based on shared OTUs and performed the principal component analysis (PCA) and 3D-principal coordinate analysis (3D-PCoA) (Figure 1). Served as another proof, Venn diagram evidenced the modulatory effect of KS on gut microbiota as different groups of treated mice have its own unique OTUs that are not shared with negative controls (Figure 1A,B). Moreover, as demonstrated in PCA and 3D-PCoA score plot, the male microbiota showed a much more significant structural shift than the female microbiota both along the first principle component and the second principle component (Figure 1C,D). This further proved that the male gut microbiota was more vulnerable than the female one in response to KS intervention. Thus, altogether, we demonstrate a sex-specific effect of KS on gut microbiota in Kunming mice.

Previously, we have identified a similar sex-dependent effect of chondroitin sulfate and its oligosaccharides on gut microbiota [30]. Here in the present study, the effect of KS on gut microbiota was also found to be sex-specific. Accumulating evidence has illustrated that sex hormones play a pivotal role in modulating the composition of gut microbiota [37]. Additionally, individual diet has also been documented to exert a sex-dependent effect on vertebrate gut microbiota [38]. Therefore, in line with previous research, it is reasonable that differences in hormone composition dictate the sex-specific effect of KS on gut microbiota. Collectively, since gut microbiota plays a critical role in host physiology [8,10,22], our study highlights that potential therapies should be tailored according to host sex during the treatment of gut microbiota-associated disease using KS.

### 2.2. Dietary KS-Modulated Gut Microbiota at Different Taxonomic Levels and Significantly Increased the Abundance of Lactobacillus *spp.*

**Figure 2:**
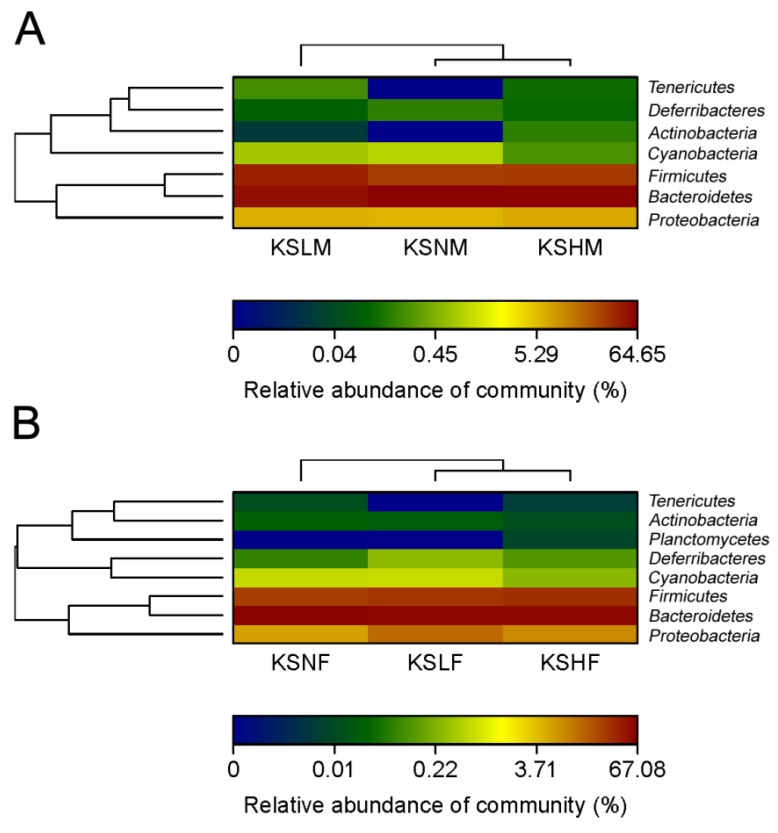
Heatmap demonstrating relative abundance of the dominant bacterial phyla in male (**A**) and female (**B**) groups.

Given that KS changed the overall structure of intestinal microbiota, the bacterial populations in each group were then compared at the phylum level. As revealed by Heatmap, both female microbiota and male microbiota were dominated by Firmicutes, Bacteroidetes and Proteobacteria (Figure 2). However, the bacteria in the three phyla respond dissimilarly to KS treatment in males and females. In male mice, the dosage groups both underwent a decrease in the abundance of Bacteroidetes and an increase in the amount of Firmicutes (Figure 2A and Figure S1). Nonetheless, in female mice, a slight increase in the abundance of Proteobacteria combined with a medium decrease in the amount of Bacteroidetes was observed after KS treatment (Figure 2B and Figure S1).

**Figure 3:**
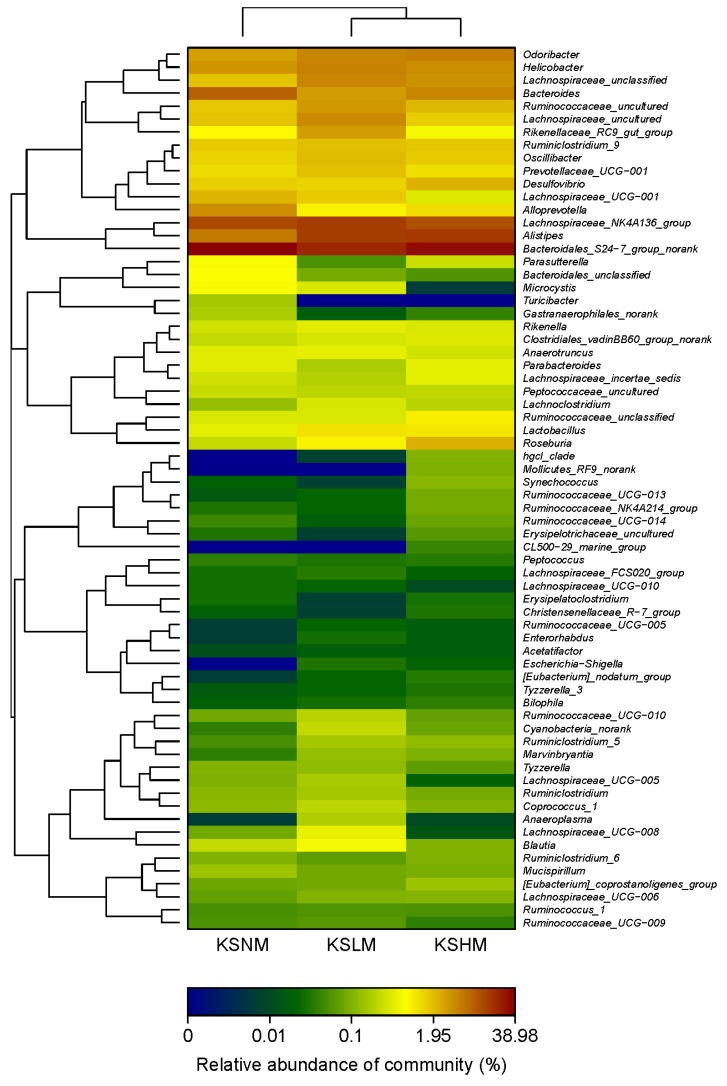
Heatmap indicating genus-level changes of the gut microbiota in male mice after KS treatment.

**Figure 4:**
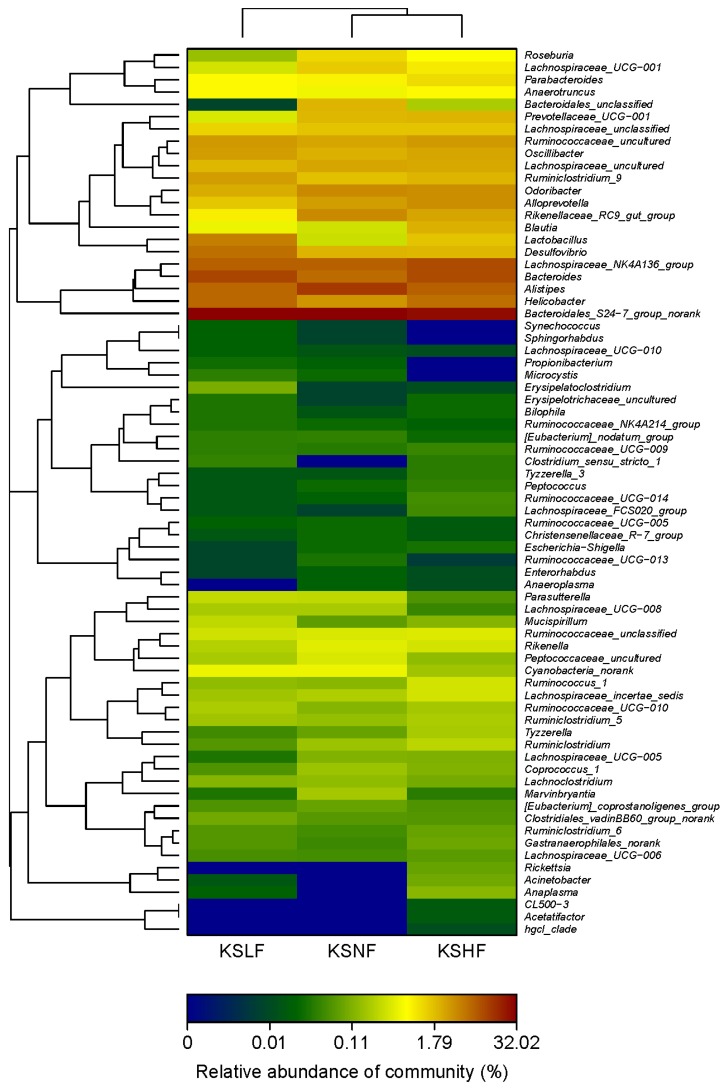
Heatmap indicating genus-level changes of the gut microbiota in female mice after KS treatment.

To further dissect the effect of KS on gut microbiota, we analyzed the bacterial communities at the genus level. As indicated by the heatmap, the gut microbiota in male mice was dominated by *Bacteroidales S24-7 group norank*, *Alistipes* spp., *Lachnospiraceae NK4A136 group* and *Bacteroides* spp. (Figure 3 and Figure S2). Compared to the KSNM group, the KSLM group has a higher amount of *Alistipes* spp. and *Lachnospiraceae NK4A136 group* but a lower abundance of *Bacteroidales S24-7 group norank* and *Bacteroides* spp. However, in the KSHM group, only *Alistipes* spp. was enriched by KS treatment. In female microbiota, two significant changes in the above four genuses, which were also the dominant groups, were the decrease of *Alistipes* spp. and increase of *Bacteroides* spp. (Figure 4 and Figure S2). Together, our results indicated that *Alistipes* spp. and *Bacteroides* spp. respond oppositely to KS treatment in male and female mice. This is in accordance with the PCA results and can be taken as a further proof for the sex-dependent effect of KS on gut microbiota.

**Figure 5:**
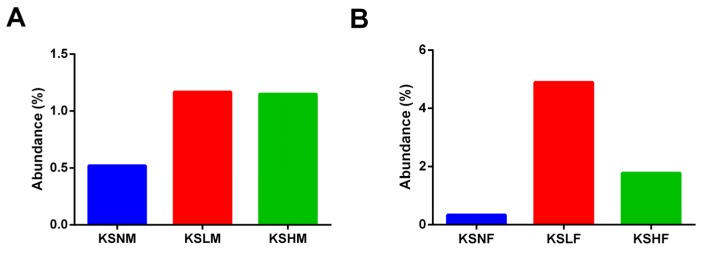
Oral intake of KS remarkably increased the abundance of *Lactobacillus* spp. in male (**A**) and female mice (**B**).

Among the genuses that were significant for the function of gut microbiota, it was remarkable to observe that the abundance of *Lactobacillus* spp. was considerably increased by KS treatment in both male and female mice (Figure 5). Furthermore, it was of interest to find that different extent of increase was achieved by KS intervention. In male mice, both low- and high-dosage of KS increased the abundance of *Lactobacillus* spp. by only one-fold (Figure 5A). However, in contrast, a sixteen-fold and a six-fold increase of *Lactobacillus* spp. were, respectively, obtained by low- and high-dosage of KS treatment in female groups (Figure 5B). Altogether, consistent with the fact that KS exerts a sex-dependent impact on gut microbiota, these observations indicate a more specific prebiotic effect of KS on gut microbiota of male and female mice.

*Lactobacillus* spp. is a genus of common probiotics that have been widely used in food and pharmaceutical industry [39,40,41]. Besides, many strains of *Lactobacillus* have been successfully applied to attenuate obesity [42,43,44], diabetes [45,46,47], diarrhea [48,49,50], and many other diseases [51,52,53] which are associated with gut dysbiosis. In this regard, since dietary KS significantly promoted the growth of *Lactobacillus* spp., further studies are therefore warranted to elucidate whether KS as a new dietary prebiotic could be used to alleviate the aforementioned diseases.

More importantly, our results can also have implications for understanding the beneficial effects of shark cartilage on osteoarthritis [54,55,56]. For more than 20 years, there has been documentation in the literature suggesting shark cartilage could be used to treat or prevent arthritis [54,55,56]. However, years passed, although different mechanisms have been proposed [56,57], and there are still questions as to how the bioactive substances from shark cartilage inhibit inflammation and attenuate symptoms of this degenerative disease. Recently, the abundance of *Lactobacillus* spp. was found to be negatively correlated with osteoarthritis [58]. Coupled with the well-documented anti-inflammatory property of *Lactobacillus* spp. [51,52,53], the prebiotic effect of KS demonstrated in the present study indicates that shark cartilage containing KS might have *Lactobacillus* spp. as its primary drug target during alleviation of osteoarthritis. Actually, to answer this question, more detailed work is now under progress in our lab.

### 2.3. KS Treatment Reduced Body Weight and Maintained Normal Gut Homeostasis

Apart from *Lactobacillus* spp., other gut bacteria including *Helicobacter* spp., *Odoribacter* spp., *Alloprevotella* spp. and *Desulfovibrio* spp. were also differentially modulated by KS treatment in male and female mice (Figure 3 and Figure 4). Taking into account that different microbes exert its own influence, regardless of good or bad, on host physiology [8,10,22] it is, therefore, of significance to elucidate whether dietary KS induced any unfavorable changes in the host after modulating the gut microbiota.

**Figure 6:**
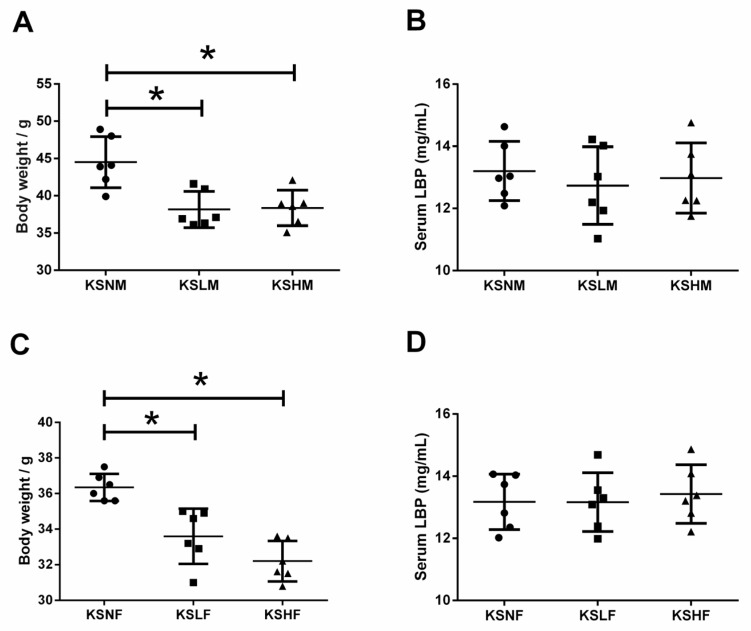
Body weight and serum LBP levels of male (**A**,**B**) and female (**C**,**D**) mice groups. The results are shown as the mean ± standard deviation. * *p* < 0.05 versus the control group.

The gut microbiota is composed of Gram-negative and Gram-positive bacteria and both of these microorganisms could trigger inflammation in the host once gut symbiosis is disturbed. Lipopolysaccharide (LPS) is an endotoxin that is only produced by Gram-negative bacteria, which, once entered the circulation system, can stimulate inflammation with the most potent capability [59,60]. Similarly, Gram-positive bacteria could also produce antigens that would cause side effects on host immunity [59,60]. As an acute phase protein that specifically binds to LPS and antigens from Gram-positive bacteria, LPS-binding protein (LBP) represents a perfect biomarker for antigen load from the gut microbiota and inflammatory response in the host [30,59,60]. Hence, to give an overall evaluation on potential harmful effect of KS during modulation of gut microbiota, the serum LBP levels in different groups of mice were determined. Remarkably, no significant changes in serum LBP levels were observed among the KS treated groups (Figure 6B,D). This indicates that dietary KS has no side effects on host physiology and maintains normal gut homeostasis during modulation of gut microbiota. However, as a dietary fiber, KS did induce a decrease in body weight (Figure 6A,C). This is in accordance with the prebiotic effect of KS because *Lactobacillus* spp., an important group of short chain fatty acid (SCFA) producers, could significantly increase the abundance of SCFAs in the gut and regulate energy homeostasis by promoting satiety [61]. 

Toll-like receptors (TLRs) are a family of evolutionary conserved receptors which play a pivotal role in host defense against invading pathogens [62,63]. TLRs recognize specific molecular patterns of microbial components including lipoprotein, lipopolysaccharides, and bacterial DNA [62,63]. Stimulation of TLRs by these factors leads to the activation of innate immunity [62,63]. Previous studies have demonstrated that certain GAGs, such as hyaluronan oligosaccharide, can bind with TLRs and activate dendritic cells [64]. However, as for KS, it could specifically block the interaction of bacterial flagellin with TLRs and suppresses the production of inflammatory cytokines [65]. This indicates that by binding with TLRs, KS would not trigger inflammation in the host. In fact, KS is potent inhibitor of inflammation in osteoarthritis when given intraperitoneally [66]. Altogether, given that shark cartilage has been used for years worldwide, as a dietary fiber from this nutraceutical, KS is perfectly safe with no obvious side effects on host immunity.

Our study has two limitations. First, as we pooled the DNA samples before sequencing, it is inevitable that information about inter-animal variations was lost. Although studies using pooled DNA samples for sequencing were still acceptable [67,68], we anticipate that future studies would sequence the gut microbiota of each mouse to further explore the prebiotic effect of KS. Second, due to the experimental design, we only focused on the effects of KS on gut microbiota. Therefore, since we did not track the food intake of the animals, we cannot tell whether the weight loss is a result of the decreased food intake or a changed structure of the gut microbiota. To address this issue, further studies are encouraged.

In summary, we first demonstrate a prebiotic activity of KS from shark cartilage using in vivo models. And in accordance with the sex-specific impact of KS on gut microbiota, the prebiotic effect of this molecule was found to be more pronounced in female mice than in males. Our current results indicate that KS can be potentially used as a novel prebiotic candidate for disease management, which merits further investigation.

## 3. Materials and Methods

### 3.1. Materials and Reagents

KS was isolated and purified from chondroitin sulfate (CS) using the method adapted from Galeotti et al. [69]. The CS extracted from shark cartilage containing KS was purchased from Rusan Wantongming Co. Ltd. (Weihai, China). The molecular weight and sulfate content of KS were determined to be 45.98 kDa and 26.06% using the protocols previously described [70]. All other chemicals of analytical grade were obtained from Sigma (Shanghai, China) unless otherwise stated.

### 3.2. Animals and Treatment

All animal experimental procedures used in the present study were approved by the Ethical Committee of Ocean University of China and complied with the National Guidelines for Experimental Animal Welfare (China, 2006) and the Guide for the Care and Use of Laboratory Animals [71]. Briefly, a total of 36 specific pathogen-free Kunming mice (six-weeks old, 18 male and 18 female) were purchased from Vital River Laboratory Animal Technology Co. Ltd. (Beijing, China). All animals were free of the following pathogens: *Pasteurella pneumotro pica*, *Klebsiella pneumonia*, *Staphylococcus aureus*, *Streptococcus pnemoniae*, *β-hemolyticstre ptococcus*, *Pseudomonas aeruginosa*, *Helicobacter pylori*, Pneumonia Virus of Mice, Reovirus type III, Minute Virus of Mice, Theiler’s Mouse Encephalomyelitis Virus, Mouse Adenovirus and Polyoma Virus. During the experimental session, all mice were housed in a controlled environment (12 h daylight cycle, lights off at 6 p.m.) in groups of three mice per cage, and kept with free access to the same batch of standard laboratory diet (M01-F, Slacom, Shanghai, China) and autoclaved fresh water. The diet is composed of 9.2% water, 22.1% casein, 5.28% lard, 9.32% cellulose, 24.08% corn starch, 23.9% sucrose, 1.24% calcium carbonate, 0.92% potassium citrate, 1.34% l-cystine, 0.95% vitamin mix, 0.95% mineral mix, and 0.72% methionine. After one-week adaptation, all animals were randomly assigned to six groups (*n* = 6 per group): Male control group (KSNM), male low-dosage group (KSLM), male high-dosage group (KSHM), female control group (KSNF), female low-dosage group (KSLF), female high-dosage group (KSHF). The control groups were given normal saline. The treated groups were given either a low-dosage (8 mg/kg) or a high-dosage (40 mg/kg) of KS by gavage. After six weeks of treatment, all mice were sacrificed by cervical dislocation. Blood samples were collected after a 12 h fasting period and centrifuged at 12,000× *g* for 20 min to pellet the blood cells. The serum lipopolysaccharide (LPS)-binding protein (LBP) levels were determined using a commercial ELISA kit (Cell Sciences, Canton, OH, USA) according to the manufacturer’s instructions. The cecal contents in each mouse were collected aseptically and stored at −80 °C before being analyzed.

### 3.3. DNA Extraction and High-Throughput Sequencing

The metagenomic DNA from each cecal sample was extracted using a QIAamp DNA Stool Mini Kit (Qiagen, Hamburg, Germany) according to the manufacturer’s instructions. The DNA concentration was quantified using a Nanodrop ND-2000 UV-VIS spectrophotometer (Thermo Scientific, Wilmington, NC, USA) and the quality of DNA was checked by gel electrophoresis. Based on different treatment protocols, the DNA samples in the same group were pooled at equimolar concentrations to generate six samples before being analyzed [30,67,68]. A pair of universal primers (338F 5′-ACTCCTACGGGAGGCAGCA-3′, 806R 5′-GGACTACHVGGGTWTCTAAT-3′) was used to specifically amplify the V3-V4 hypervariable regions of the 16S rRNA gene. The PCR reactions were conducted in a thermocycler PCR system (GeneAmp 9700, ABI, Foster City, CA, USA) using the method described elsewhere [30]. After amplification, the PCR products were checked and purified by an AxyPrep DNA Gel Extraction Kit (Axygen Biosciences, Union City, CA, USA) according to the manufacturer’s specifications. The purified amplicons were then quantified by QuantiFluor-ST (Promega, Madison, WI, USA) and sequenced under the MiSeq platform (Illumina, San Diego, CA, USA) by a commercial company (Majorbio Bio-Pharm Technology Co. Ltd., Shanghai, China) using standard programs.

### 3.4. Bioinformatics and Analysis of the Sequencing Data

Quality control of the raw sequencing data was performed by QIIME (version 1.17) using the criteria previously described [60]. The Operational Taxonomic Units (OTUs) were clustered using UPARSE (version 7.1) pipeline [60]. The Venn diagram was constructed using R packages (version 3.1.0, R Core Team, Auckland, New Zealand) to compare the compositional OTUs in the gut microbiota of different groups. Mothur (version V.1.30.1) was applied to perform the refraction and alpha diversity analysis. To evaluate the community richness and community diversity, Chao1, Ace, Simpson, and Shannon indices were respectively calculated [72]. The structure of different microbial communities was compared by principal component analysis (PCA) and 3D-principal coordinate analysis (3D-PCoA) [59,60]. Taxonomical assignments of each sequencing reads were performed by a RDP classifier and the heatmap was constructed to illustrate the compositional differences of each microbial community at both phylum and genus levels [59].

### 3.5. Statistical Analysis

Data for the body weight and serum LBP levels are expressed as the mean ± standard deviation. Statistical analyses for the body weight and serum LBP levels were performed by one-way ANOVA and Bartlett’s test (SPSS Software 12.0, North Chicago, IL, USA). All results were considered statistically significant at * *p* < 0.05 versus the control group.

## Figures and Tables

**Table 1:** Diversity of gut microbiota in control and KS-treated mice.

Groups	Reads	OTUs	Good’s Coverage	Richness Estimator		Diversity Estimator	
				Ace	Chao1	Shannon Indices	Simpson Indices
KSNF	19719	247	0.999036	256	256	4.39	0.0201
KSLF	18384	248	0.998749	260	262	4.24	0.0280
KSHF	25456	263	0.999568	268	266	4.45	0.0220
KSNM	28432	254	0.999402	262	263	4.45	0.0188
KSLM	24572	255	0.999390	262	268	4.42	0.0227
KSHM	32141	258	0.999720	261	261	4.41	0.0209

Abbreviations: female control group (KSNF), female low-dosage group (KSLF), female high-dosage group (KSHF), male control group (KSNM), male low-dosage group (KSLM), and male high-dosage group (KSHM).

## Supplementary Materials

The following are available online at www.mdpi.com/1660-3397/14/12/s1, Figure S1: Structural comparison of gut microbiota at the phylum level; Figure S2: Structural comparison of gut microbiota at the genus level.
