# Supplementary material for: Dietary Keratan Sulfate from Shark Cartilage Modulates Gut Microbiota and Increases the Abundance of Lactobacillus spp
Source: Mar Drugs. 2016 Dec 8;14(12):224. doi: 10.3390/md14120224 (PMC5192461; doi:10.3390/md14120224)
Supplement: Supplementary file 1 [file marinedrugs-14-00224-s001.doc]

Supplementary Materials: Dietary Keratan Sulfate from Shark Cartilage Modulates Gut Microbiota and Increases the Abundance of *Lactobacillus* spp.

Qingsen Shang, Qinying Li, Meifang Zhang, Guanrui Song, Jingjing Shi, Hao Jiang, Chao Cai, Jiejie Hao, Guoyun Li and Guangli Yu


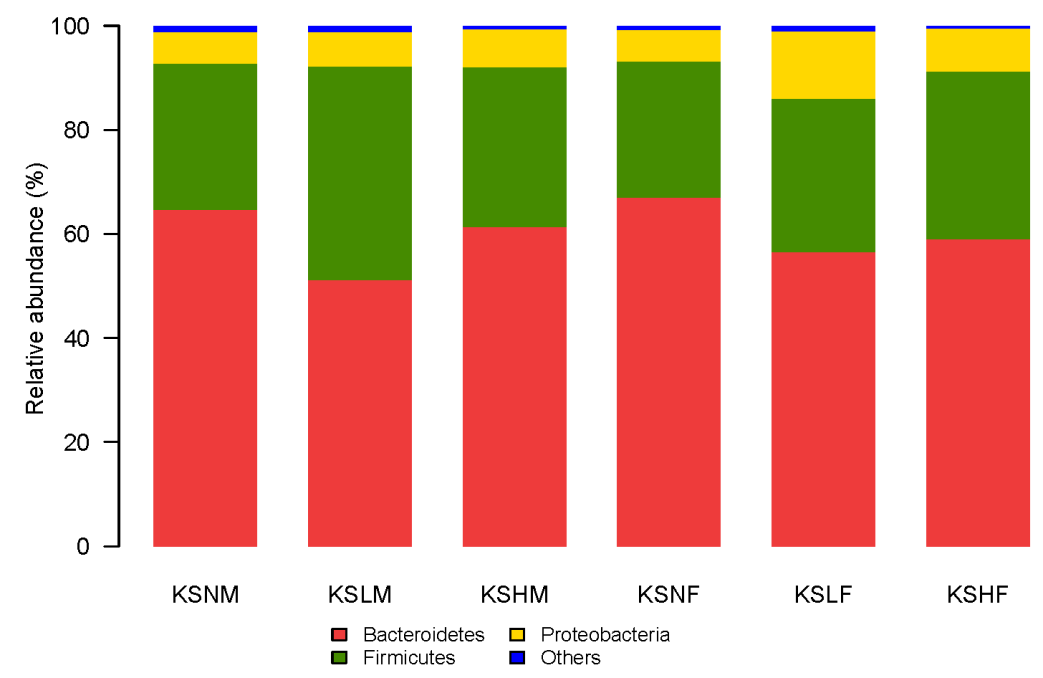


**Figure S1.** Structural comparison of gut microbiota at the phylum level.


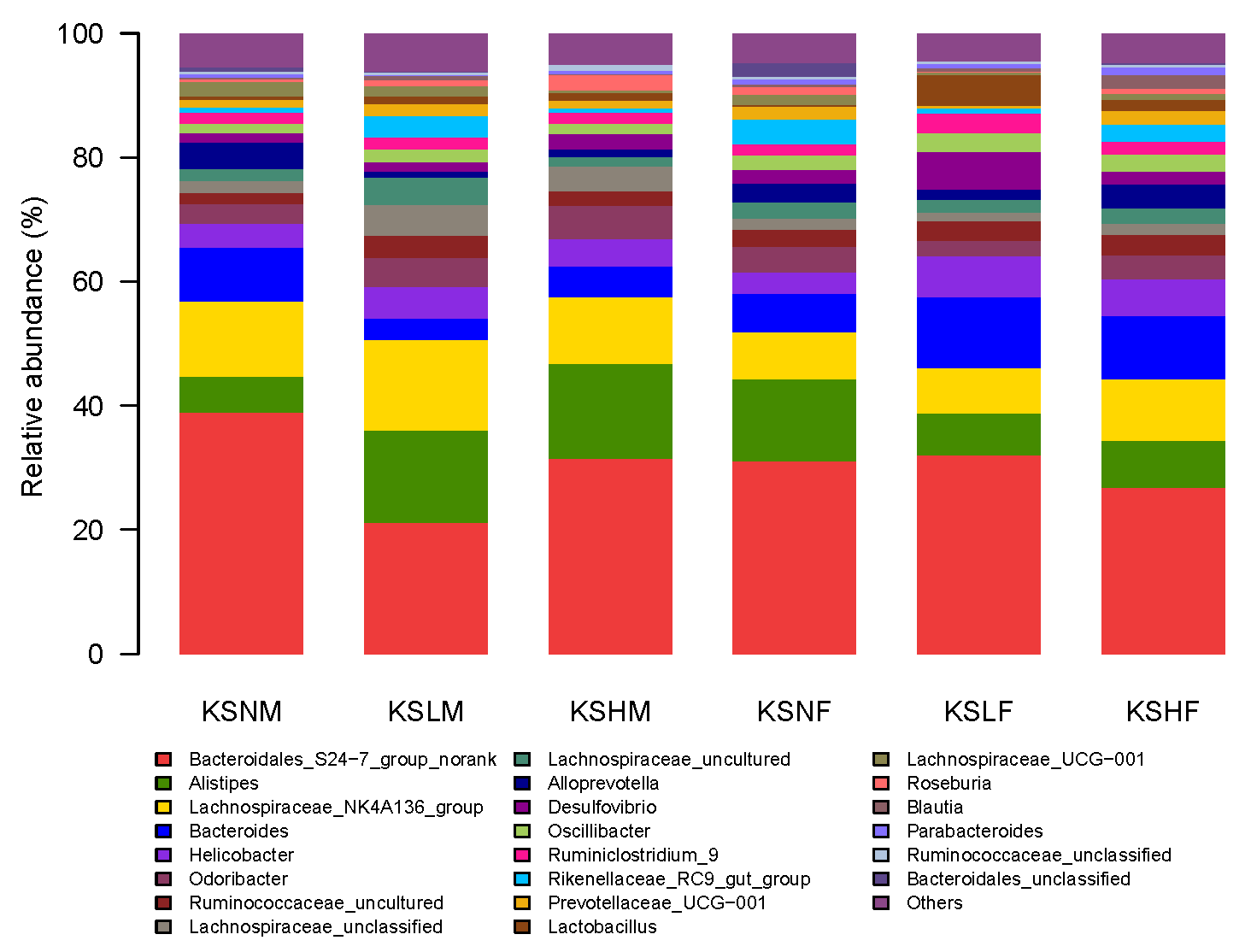


**Figure S2.** Structural comparison of gut microbiota at the genus level.
